# Nerve root compression due to lumbar spinal canal tophi: A case report and review of the literature

**DOI:** 10.1097/MD.0000000000031562

**Published:** 2022-11-11

**Authors:** Kai Wang, Quan-Zeng Yang, Hao-Nan Wen, Yun-Xaing Hai, Guo-Dong Gao, Min Song

**Affiliations:** a Clinical College of Chinese Medicine, Gansu University of Chinese Medicine, Lanzhou, Gansu Province, China; b Department of Orthopedics, The Affiliated hospital of Gansu University of Chinese Medicine, Lanzhou, Gansu Province, China.

**Keywords:** case report, gout, literature review, lumbar spine, uric acid

## Abstract

Gout in the spine and adnexa is rare in clinical practice and can also be easily misdiagnosed, we reported a patient with nerve root compression due to lumbar gout stones in the lumbar spinal canal.

**Patient concerns:**

A 51-year-old male was admitted to the hospital with lumbar pain with numbness in the left lower limb for more than 6 months. The physical examination showed that tenderness and percussion pain were present at L4-S1 spinous process. Straight leg raise test: 50° on the left side were positive. Laboratory tests showed that the sUA was 669 μmol/L, MRI of the lumbar spine showed that cystic T1WI low signal and T2WI mixed high signal shadows were seen in the spinal canal at the level of L4-L5.

**Diagnoses:**

Combining with lab examinations, imaging examinations, and histopathological results, the patient was diagnosed with lumbar spinal canal tophi.

**Interventions:**

After active improvement of all examinations, the patient underwent surgical treatment with decompression and internal fixation of the L4-L5 segment.

**Outcomes:**

After surgery, the patient’s symptoms improved and muscle strength returned to normal. Among the 95 previously reported patients with lumbar gout, the ratio of men to women was 2.96:1, and the peak age group of incidence was 56 to 65 years. The onset of the disease was mainly in a single segment of the lumbar spine, with 34.41% of all cases occurring at the L4-L5 level. 61.05% of the patients had a history of gout attacks or hyperuricemia, and the most frequently involved site was the foot and ankle, followed by the wrist. Sixty-seven patients underwent surgical treatment, and 22 chose conservative treatment, with overall satisfactory results.

**Lessons subsections:**

The incidence of lumbar gout is low and relatively rare in the clinic and pathological biopsy is still the gold standard. Vertebral plate incision and decompression are often selected for surgical treatment, and whether to perform fusion should be comprehensively considered for the destruction of vertebral bone by gout and the reasonable selection of the extent of surgical resection. Whether choosing surgical treatment or conservative therapy, the control of uric acid levels should be emphasized.

## 1. Introduction

**Figure 1.**
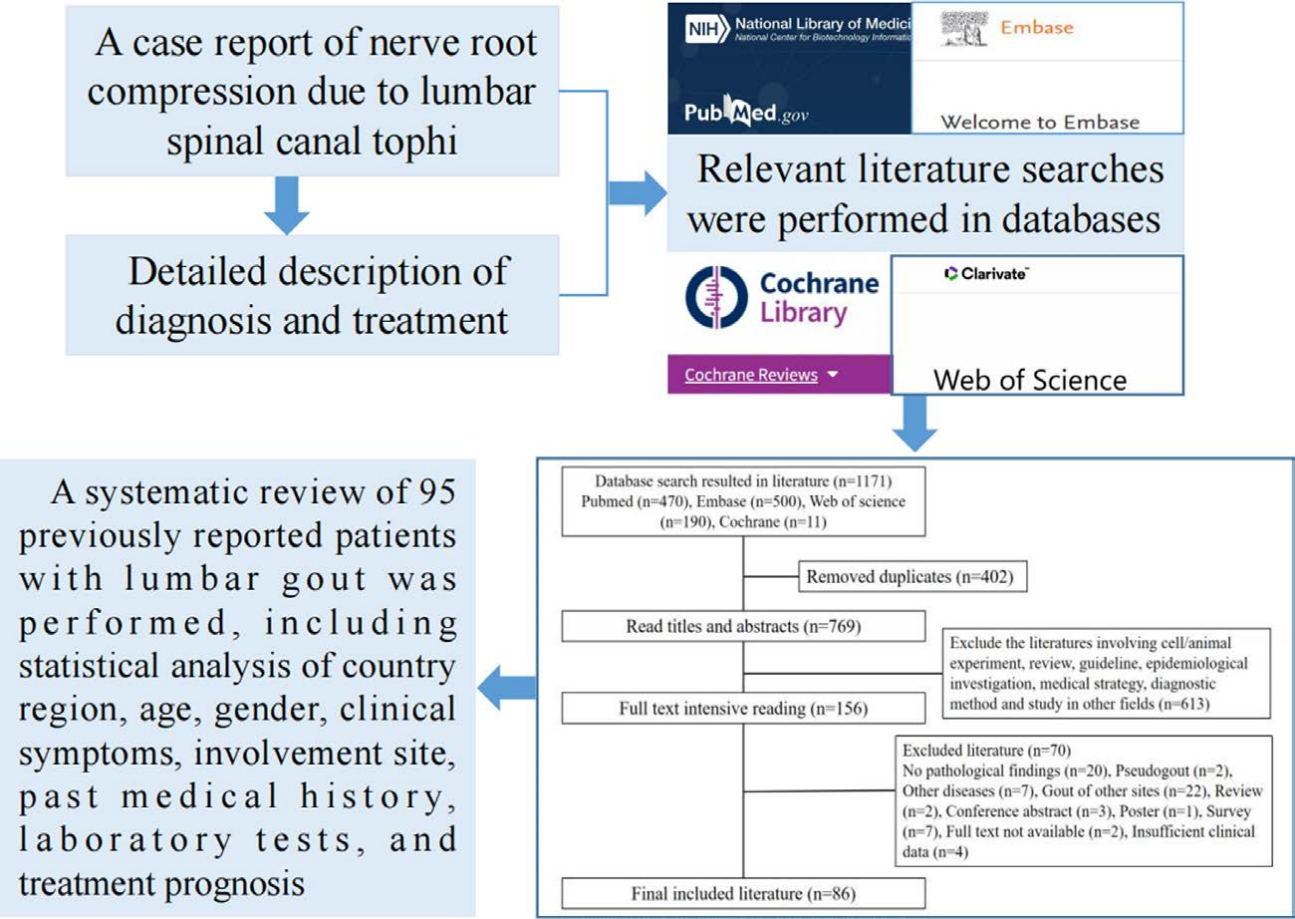
The workflow diagram.

Gout is a systemic metabolic disease caused by disorders of purine metabolism and impaired uric acid excretion, characterized by recurrent acute and chronic arthritis, joint deformity and severe pain, with increased blood uric acid and deposition of urate crystals in joints, ligaments, tendons, subcutaneous and other sites causing tissue damage as its most important pathological basis.^[1]^ Gout is seen all over the world, with a global prevalence of 1% to 4% and an incidence of 0.1% to 0.3%. With rapid economic development and continuous improvement in quality of life, the prevalence of gout in China has risen sharply to 1% to 3%,^[2]^ and is trending younger, greatly increasing the risk of cardiovascular disease and seriously threatening the physical and mental health of patients. The first attack of gout usually involves a joint, most commonly the first metatarsophalangeal joint, followed by small joints of the extremities, more in the lower extremities than in the upper extremities, and gout in the spine and adnexa is rare, In particular, gout stones in the lumbar spinal canal causing obvious nerve root compression symptoms are much rarer in clinical practice and are difficult to differentiate from lumbar disc herniation, lumbar spinal stenosis, intervertebral space infection and other occupying lesions, making early diagnosis and treatment difficult. But due to the bone connection structure of the spine, urate can also be deposited in the vertebrae, synovial joints, intervertebral discs, ligamentum flavum, interspinous and supraspinous ligaments, and other tissues where gout occurs. The incidence of gout in the lumbar spine is approximately 56%, and 22% in each of the cervical and thoracic spines.^[3]^ Spinal gout was first reported by Kersley et al in 1950,^[4]^ and in recent years gout stones involving the cervical, thoracic, and lumbar spinal canal and beyond have been reported both at home and abroad, but due to the limited amount of literature and the fact that they are all case reports, clinicians are not sufficiently aware of spinal gout. We report a patient with gout stone deposited in the lumbar spinal canal causing nerve root compression and review the literature related to lumbar gout. We searched the relevant literature in the database to analyze the country region, age, gender, clinical manifestations, statistically sites, past medical history, and treatment prognosis of patients with lumbar gout in order to improve clinicians’ understanding of the disease. The workflow is shown in Figure 1.

## 2. Case report

This 51-year-old male patient visited our hospital on December 27, 2018 due to lumbar pain complicated with numbness of left lower limb for more than half a year and aggravation for 1 day. Six months ago, the patient developed lumbar pain without any apparent cause, accompanied by radiating pain and numbness in the left lower limb, which seriously affected her daily life and was aggravated by exertion and cold, especially at night and could be relieved after rest. The symptoms were alleviated after infusion treatment in a community hospital, but the specific medication and dosage were unknown. One day before admission, he was admitted to the hospital for further treatment because of significantly increased lumbar pain after overexertion, unfavorable lateral rotation, and significantly limited functional activity. The patient had a past history of gouty arthritis for 2 years, which was not treated formally; he had a history of hypertension for ten years, with a maximum of 180/140 mm Hg, which was treated with oral telmisartan tablets and indapamide extended-release tablets, and was now 104/81 mm Hg, which was still under control. He denied family history, history of coronary heart disease, diabetes mellitus, and history of infectious diseases such as hepatitis and tuberculosis. Since the onset of the disease, the patient had normal appetite, poor sleep at night, regular bowel movements and no significant change in weight.

The physical examination showed that the spinal sequence was normal, without scoliosis and retroflexion, the lumbar physiological curvature existed, and the lumbar muscles were tense and spastic. Tenderness and percussion pain were present at L4-S1 spinous process and 1.5 cm on both sides of spinous process, and there were no radiological symptoms of the lower extremities when percussion was performed. The jerk test was positive. Straight leg raise test: 80° on the right side and 50° on the left side were positive. The muscle strength of the quadriceps and tibialis anterior muscles of the left lower limb was grade III, and the skin sensation of the first metatarsal and dorsal toe was decreased. The peripheral blood flow of both lower limbs was normal, the physiological reflexes were normal, and the pathological reflexes were not elicited. There was no sign of gout stone deposition in peripheral joints and other areas.

**Figure 2.**
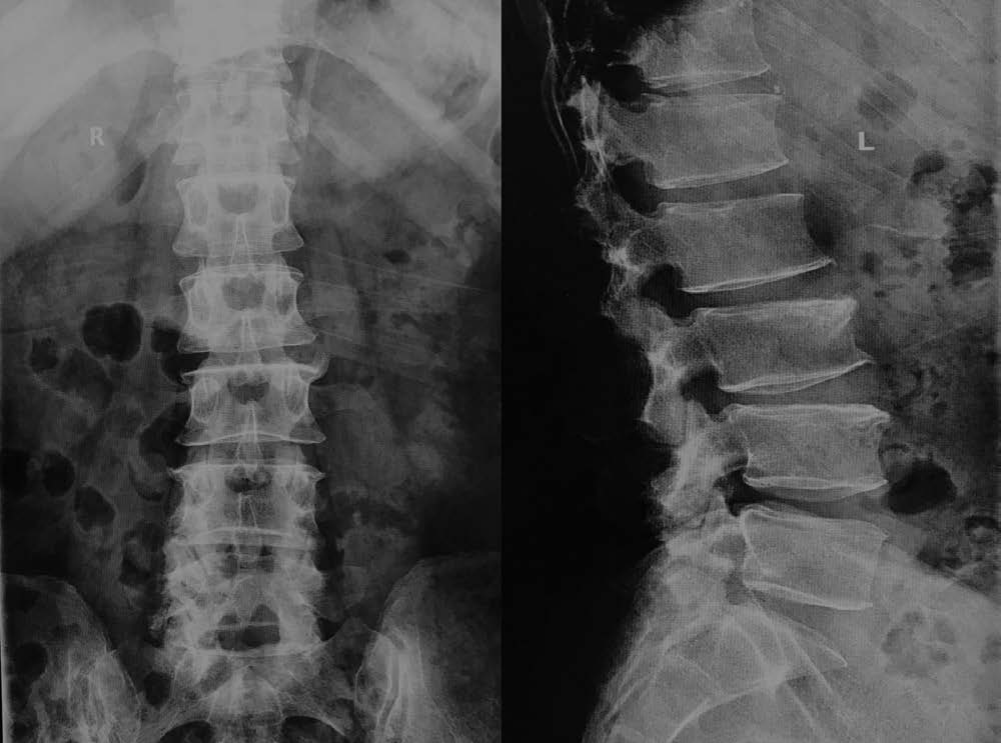
X-ray of the lumbar spine with no significant abnormalities except for bone formation at the vertebral body margins (ortho and lateral views).

**Figure 3.**
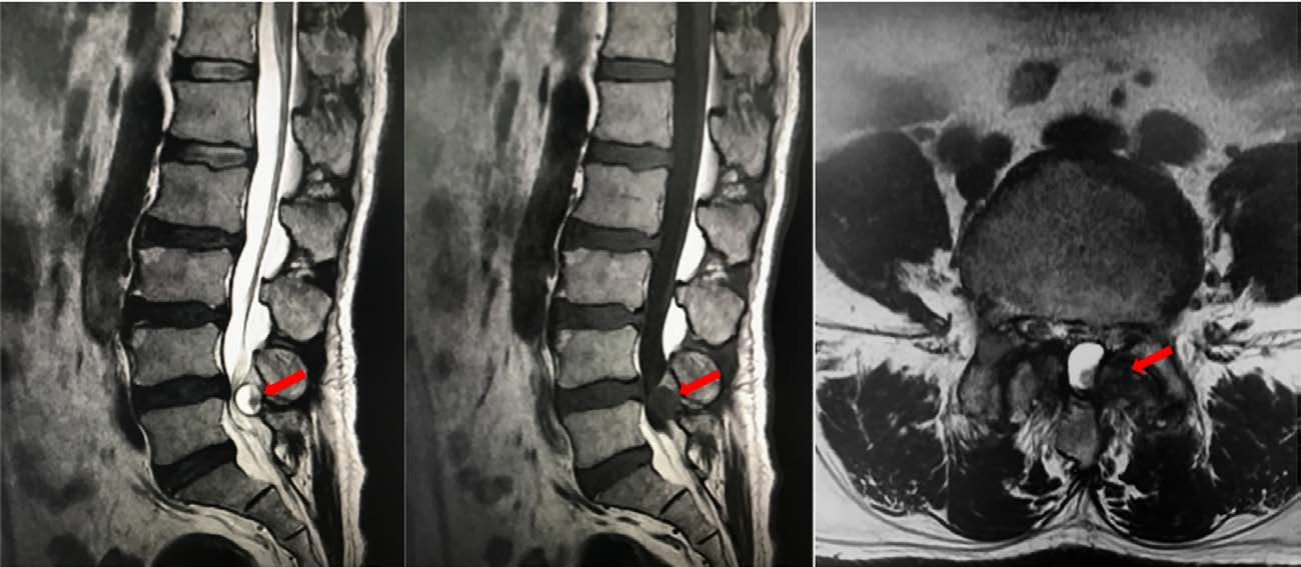
Preoperative MRI sagittal and cross-sectional views of the lumbar spine showing an occupying lesion in the spinal canal at the L4-L5 level (shown by red arrows). Note: A cystic T1WI low signal and mixed T2WI high signal shadow is seen in the spinal canal at the L4-L5 level, with adjacent nerve fibers displaced by compression. MRI = magnetic resonance imaging.

Laboratory tests showed that the patient’s serum sUA was 669 μmol/L (normal 208–428 μmol/L), triglycerides 1.76 mmol/L (normal 0–1.7 mmol/L), glutamyl aminotransferase 153 U/L (normal 7–50 U/L), total cholesterol 2.76 mmol/L (normal 2.9–5.72 mmol/L), potassium 3.38 mmol/L (normal 3.5–5.5 mmol/L), and other parameters were in the normal range. X-rays showed that the physiological curvature of the lumbar spine existed, the sequence was as normal, different degrees of hyperplasia-like changes were seen at the edges of some vertebral bodies, the vertebral body surface was smooth, and no significant abnormalities were seen in the intervertebral space and accessory structures (Fig. 2). MRI of the lumbar spine showed that cystic T1WI low signal and T2WI mixed high signal shadows were seen in the spinal canal at the level of L4-L5, and the adjacent nerve fibers were displaced by compression, and neurogenic tumors or arachnoid cysts were mostly considered (Fig. 3); the L2-L5 intervertebral disc had partially reduced signal in T2, and Stir showed low signal changes, and the L4-L5 and L5-S1 intervertebral discs also protruded into the posterior vertebral body. The dural sac was compressed, the fatty space was lost, and the intramedullary signal was not uniform.

**Figure 4.**
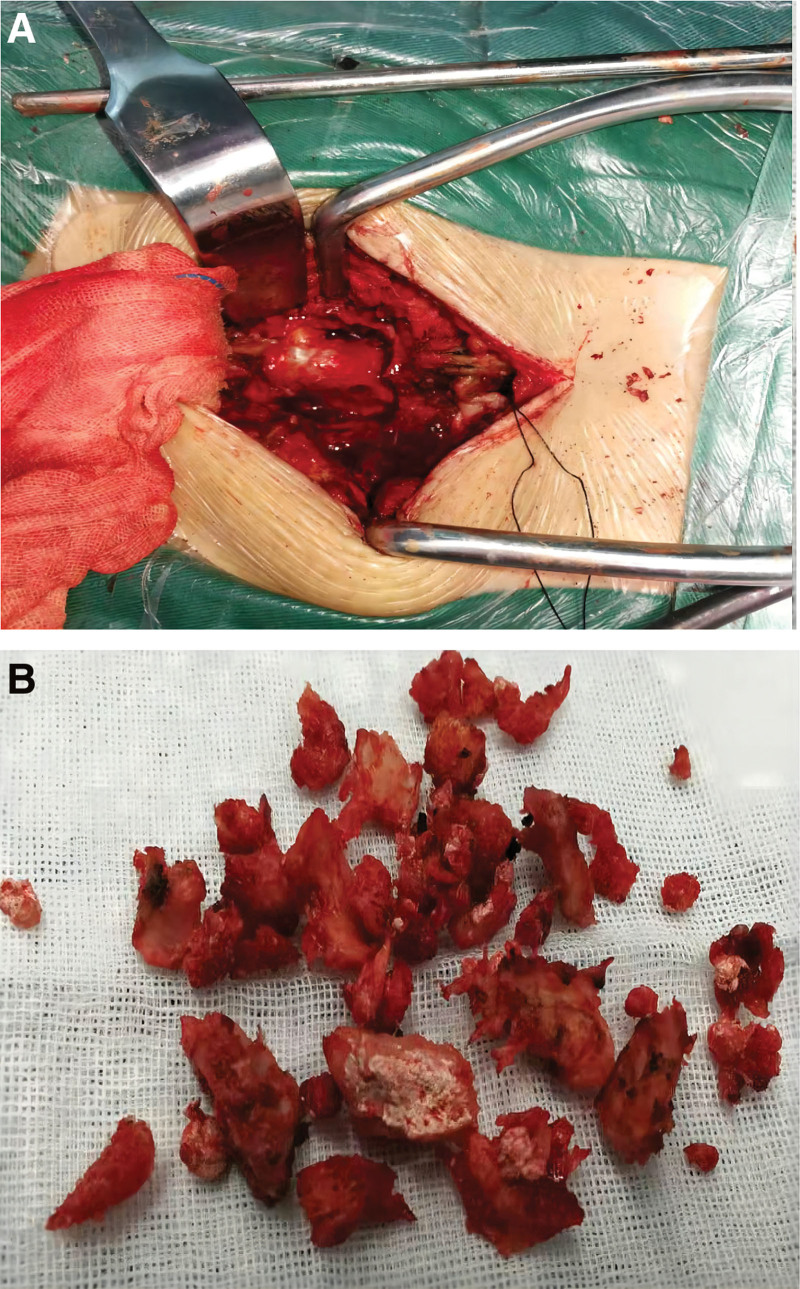
(A and B). Intraoperative cystic structure located at the dorsal aspect of the dura at the level of L4-L5 with an intact envelope that is adherent to the dura at its base and contains a yellow jelly-like substance and a plaster-like white granular substance filling the lateral crypt.

The patient was initially diagnosed with an occupying lesion at the L4-L5 level and a lumbar disc herniation. Considering the patient’s elevated uric acid level and a history of gouty arthritis for more than 2 years, it was suspected that the nature of the mass was related to a history of gout. The patient underwent decompression of the L4-L5 segment and internal fixation with the nail bar system 2 days after admission. During the operation, the lower 1/2 of the right vertebral plate of L4 was chiselled away with a bone knife, and a plaster-like white granular material was seen below the right vertebral plate of L4 and filled in the lateral saphenous fossa, the white foreign body was removed and flushed with a large amount of saline, and after probing for no residue, the vertebral plate was gradually bitten away toward the midline using a vertebral plate biting forceps, and the cystic structure located at the dorsal side of the dura at the level of L4-L5 was gradually revealed, which had an intact periosteum and was adherent to the dura at the bottom of the periosteum that contained a yellow jelly-like substance (Fig. 4A). The cyst was removed along the lateral side of the cyst wall using a nerve stripper, and the dura was also filled with plaster-like white granular material on the left side of the dura, so the lower 1/2 of the vertebral plate on the left side of L4 was chiselled away using a bone knife, and the plaster-like white granular material was removed from the lateral crypt under the left side of L4 (Fig. 4B). After flushing with a large amount of saline, there was no residue on exploration, and 4 polyaxial pedicle screws (55 × 50 mm) were sequentially impacted in the L4-L5 pedicle, and good screw position was observed on fluoroscopy, the incision was flushed with saline, and the incision was closed layer by layer after a built-in silicone drainage tube. Intraoperative bleeding was about 300 mL without blood transfusion, and the resected specimen was sent to the family for pathological examination after passing through the eyes.

**Figure 5.**
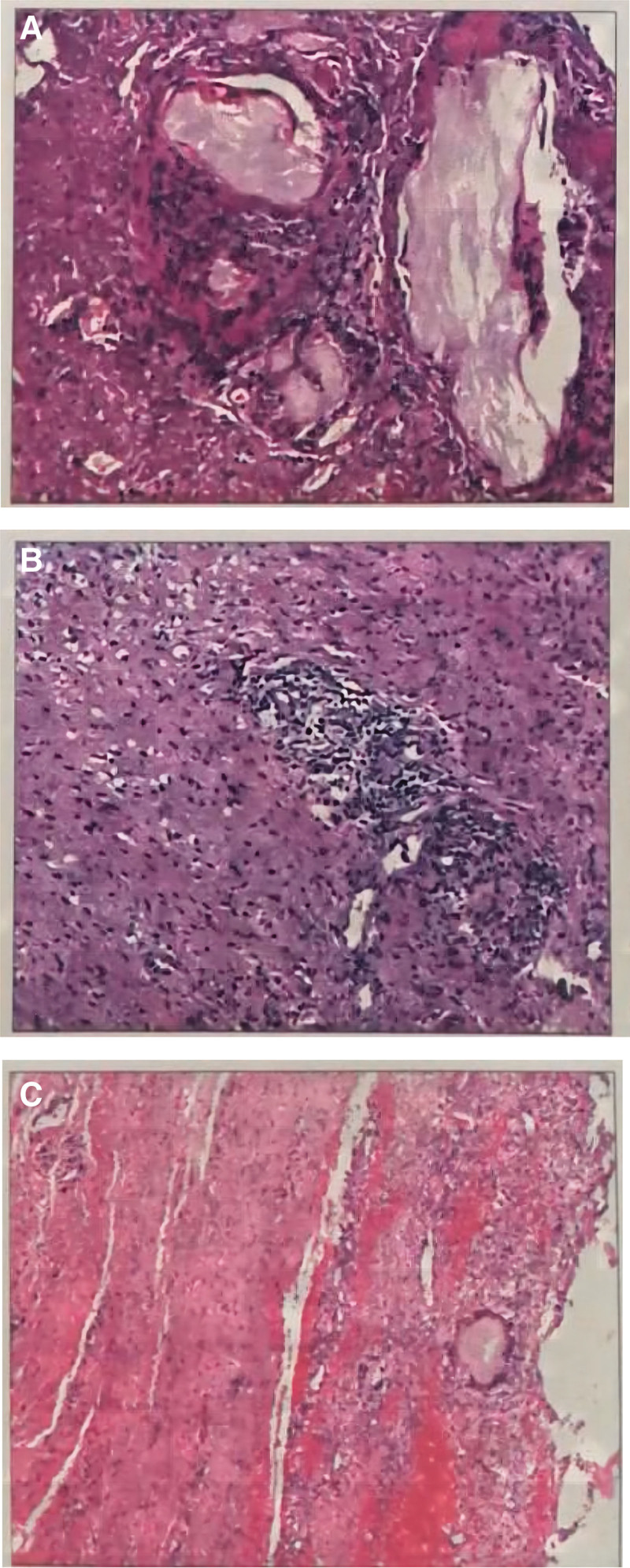
(A–C). Intradural resection of fibrous, fatty and transverse muscle tissue, with some urate crystals in the fibrous tissue with giant cell reaction, consistent with gout pathology.

**Figure 6.**
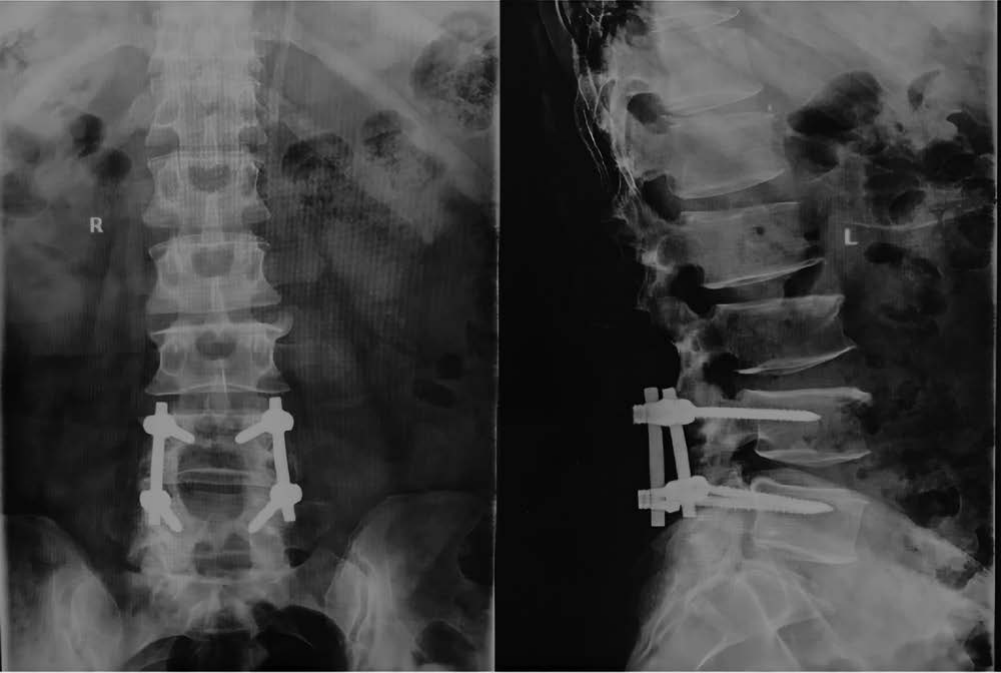
Lumbar spine X-ray showing good internal fixation position at the L4-L5 level (orthogonal and lateral views). Note: The L4-L5 vertebrae are visible with metal internal fixation, some of the corresponding vertebral plates and spinous processes are absent, multiple vertebral marginal spurs are present, the vertebral space is as normal and the paravertebral soft tissue shadow is clear.

Postoperative pathology report: gross observation saw (intravertebral canal cyst) a pile of gray-white and gray-brown broken-shaped tissue, size 5*5*1 cm; microscopically seen sent for fiber, fat and transverse muscle tissue, some urate crystals were seen within the fiber tissue with giant cell reaction; the diagnosis was consistent with the pathological changes of gout (Fig. 5A–C). After the operation, the patient’s lumbar pain symptoms improved significantly, the numbness and discomfort of the left lower limb improved significantly, he could go down normally, and his muscle strength returned to normal. Cefazolin sodium pentahydrate was given to prevent infection, mannitol and dexamethasone injection to dehydrate and reduce swelling, and febuxostat to control uric acid, and the patient was discharged ten days after surgery with normalized uric acid level (319 μmol/L). At 13-month follow-up, the internal fixation was in good position (Fig. 6), the nerve root compression symptoms had been largely relieved, and functional activities were well recovered.

## 3. Literature search

Relevant literature searches were performed in PubMed, Embase, Cochrane Library and Web of Science databases using “lumbar gout,” “lumbar spinal gout,” and “spinal gout” as keywords, and the publication time was set from database establishment to December 2021. The retrieved literature was imported into NoteExpress (version 3.2.0.7535) in “txt” text format, and the matching degree was first set to check, and only 1 duplicate literature was retained, and then manual checking was done to complete the initial screening. Read the article title and abstract, and eliminate the documents that do not meet the requirements. The target literature obtained by full-text reading was entered into Microsoft Excel 2018 software after extracting relevant clinical data information according to the inclusion and exclusion criteria. The above steps were completed independently by 2 authors, and the research team discussed and checked to ensure the accuracy and reliability of the data.

Literature inclusion criteria: patients were diagnosed with lumbar gout, including intervertebral space, vertebral adnexa and intravertebral canal; with clear pathological diagnosis by surgical resection or computed tomography (CT)-guided puncture biopsy; the type of literature was case report or clinical observational study; all patients had complete basic information and clinical data. Exclusion criteria: gout involving the cervical spine, thoracic spine, sacroiliac joints, etc; those with suspected diagnosis of lumbar gout without pathological biopsy; those belonging to other diseases such as lumbar disc herniation, lumbar metastatic tumor, tuberculosis, brucellosis spondylitis, psoriatic spondyloarthropathy, intervertebral infection, etc; Studies based on animal and cell experiments; In addition, there are literature types such as reviews, diagnosis and treatment guidelines, expert consensus, conference abstracts, posters and imaging diagnostic methods.

## 4. Analysis of results

**Figure 7.**
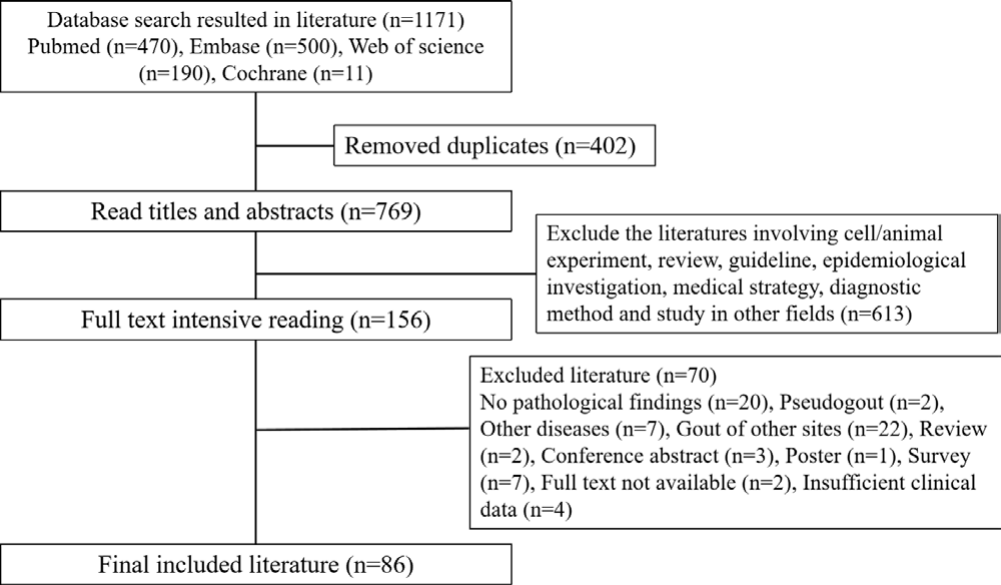
Literature search flow chart.

A total of 1171 relevant pieces of literature were retrieved, and 402 duplicate literature were removed, resulting in the initial inclusion of 769 literature. After reading the abstracts and titles, 613 irrelevant literature were excluded, the remaining 156 pieces of literature were refined for full-text re-screening, and 86 literature were finally included according to the inclusion and exclusion criteria all of which were case reports (Fig. 7). A systematic review of 95 previously reported patients with lumbar gout was performed, including statistical analysis of country region, age, gender, clinical symptoms, involvement site, past medical history, laboratory tests, and treatment prognosis.

### 4.1. Number of reported cases by country

**Figure 8.**
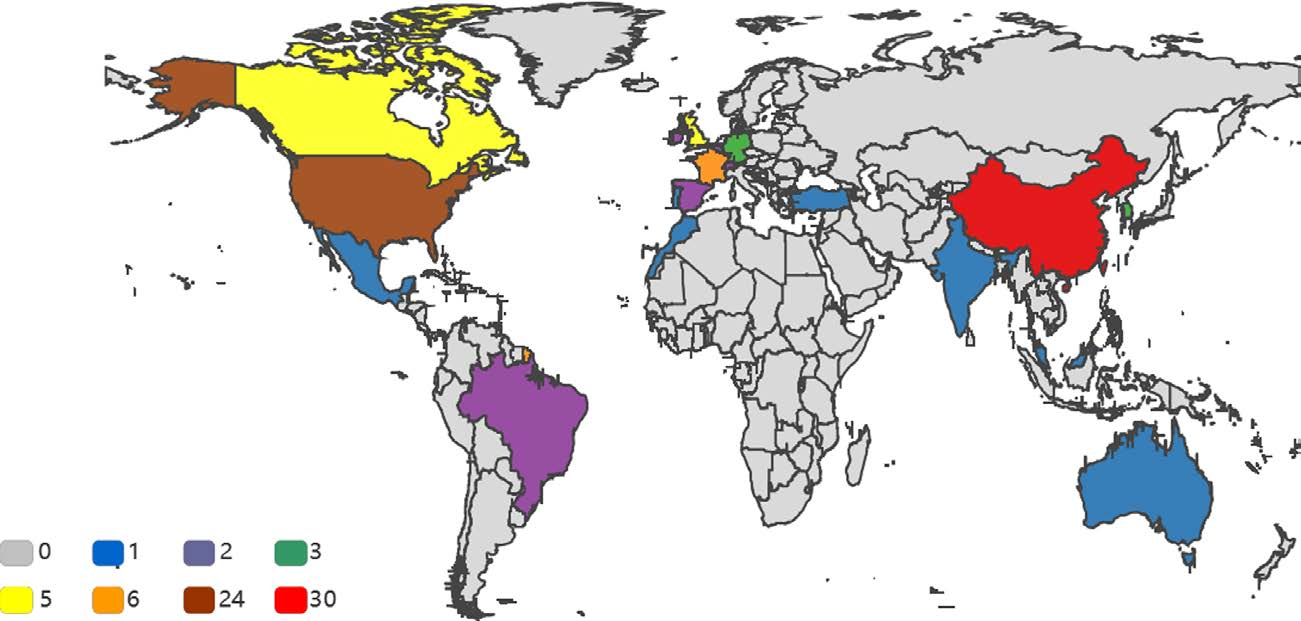
Number of case reports by country, the different colors represent the number of reports for each country.

The 95 patients were distributed in 20 different countries (Fig. 8), including 30 in China, 24 in the United States of America, 6 in France, 5 each in Canada and the United Kingdom, 3 each in Germany and Korea, 2 each in Ireland, Brazil, Belgium, Switzerland, Singapore and Spain, and 1 each in Morocco, Mexico, Portugal, Turkey, Malaysia, India and Australia.

### 4.2. Reported cases of lumbar gout in different years

**Figure 9.**
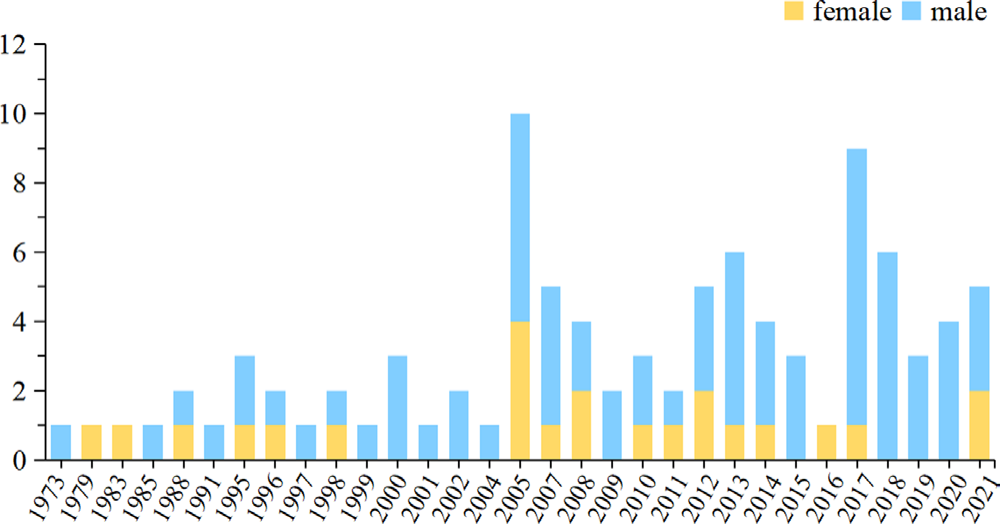
Number of reported cases of lumbar gout by year.

Among the 95 previously reported cases of lumbar gout, dating back as far as 1973, it is easy to see that the overall number of reports from 1973 to 2021 is still low, especially before 2004, when the number of reports was basically no more than 3 cases per year (Fig. 9), while the highest number of reports was 10 cases (10.53%) in 2005, followed by 9 cases (9.47%) in 2017, 6 cases reported in 2013 (6.32%), and 6 cases reported in 2018 (6.32%).

### 4.3. Gender and age distribution

**Figure 10.**
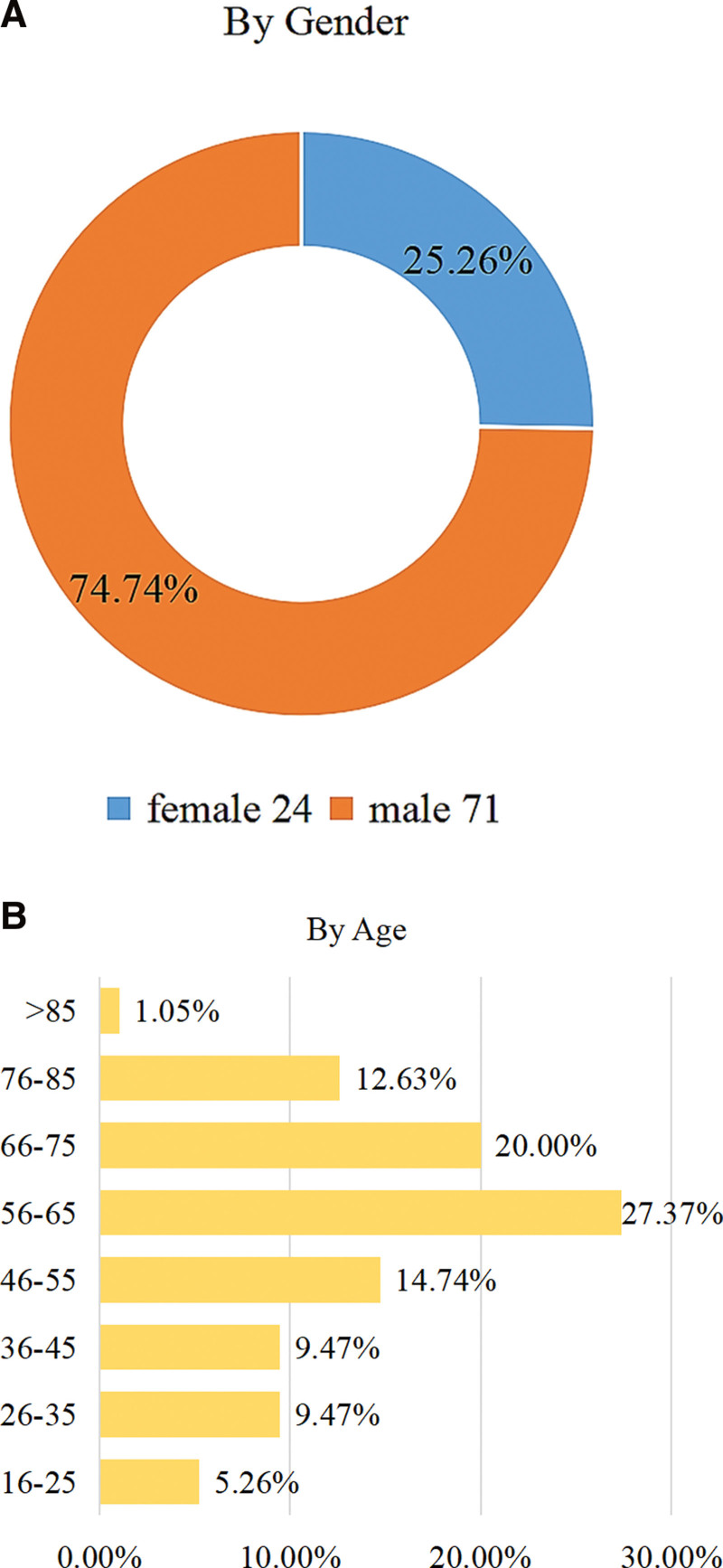
(A and B). Graphical representation of gender and age groups.

Among the 95 patients with lumbar gout, 71 cases (74.74%) were male, and 24 cases (25.26%) were female, with a male to female ratio of 2.96:1 (Fig. 10A). The highest age of the patients was 87 years old, the youngest age was 16 years old, the average age was (57.15 ± 16.80) years old, and the peak age of onset was 56 to 65 years old (Fig. 10B), and the distribution statistics by age group were 56 to 65 years 27.37% (26/95), 66 to 75 years 20% (19/95), 46 to 55 years 14.74% (14/95), 76 to 85 years old 12.63% (12/95), 36 to 45 years old 9.47% (9/95), 26 to 35 years old 9.47% (9/95), 16 to 25 years old 5.26% (5/95), 85 years or older 1.05% (1/95).

### 4.4. Past medical history and family history

Of the 95 patients, 28 cases (29.47%) had no history of gout attacks or hyperuricemia, 9 cases (9.47%) had no past medical history described, and 52 cases had gout attacks (54.73%), and 6 cases (6.32%) were hyperuricemic. Of the 28 patients who did not have gout or hyperuricemia, 11 cases (39.29%) had hypertension, 10 cases (35.71%) had obese manifestations, 4 cases (14.29%) showed only polyarticular pain, 1 case (3.57%) had hyperlipidemia, 1 case (3.57%) had hypercholesterolemia, and 1 case (3.57%) had hypercreatinemia. In 52 patients with a history of gout, 30.77% (16/52) had hypertension in combination, 23.08% (12/52) had renal function abnormalities, 4 cases (7.69%) had obesity, 3 cases (5.77%) had elevated lipids, and 1 case (1.92%) was diagnosed with metabolic syndrome; 24 cases (46.15%) did not describe the duration of gout attacks, and the duration of the remaining 28 cases ranged from 1 week to 25 years, with a peak distribution of 10 to 20 years 46.43% (13/28); 5 patients (9.62%) did not have other sites involved, and 14 cases (26.92%) did not describe the involvement of other parts. The most frequently involved site was the foot and ankle 63.64% (21/33), notably only 19.04% (4/21) involved the first metatarsophalangeal joint, followed by the wrist 48.48% (16/33), the knee 42.42% (14/33), and only 6.06% (2/33) patients presented with gout stones in the auricle. In addition, 91 (95.79%) patients did not describe whether they had a family history, 2 (2.11%) patients had a definite family history, and 2 (2.11%) patients did not have a family history.

### 4.5. Clinical symptoms

The clinical symptoms were mainly lumbago, backache, fever and different degrees of neurological dysfunction. Fifty-seven patients (60%) had symptoms of lumbago, 19 patients (20%) had symptoms of fever, 14 cases (14.74%) showed backache, 7 cases (7.37%) had symptoms of low-back pain, 6 cases (6.32%) had lumbosacral pain, 9 cases (9.47%) patients only had discomfort symptoms such as pain and numbness in the lower limbs, and 2 cases (2.11%) had only polyarticular pain as the primary clinical manifestation. There were 68 patients (71.58%) with different degrees of neurological dysfunction, 45 cases (66.18%) with radiating pain in the lower limbs, including 44.44% (20/45) in the right lower limb, 31.11% (14/45) in both lower limbs, and 24.44% (11/45) in the left lower limb; 19 cases (27.94%) with numbness, weakness, and sensory impairment in the lower limbs; 7 cases (15.56%) had intermittent claudication, 4 cases (8.89%) had gait abnormalities, and 2 cases (4.44%) showed ataxia; 5 patients (11.11%) had problems with urination and defecation, of which 60% (3/5) had diaphoresis, and 40% (2/5) had urinary incontinence.

### 4.6. Course of disease

The duration of lumbar gout was described in 17 patients (17.89%), and the duration of lumbar gout in 78 patients (82.11%) ranged from 3 days to 19 years, with a peak duration of 1 week to 1 month, and the distribution by a time period of duration was 32.05% (25/78) for 1 week to 1 month, 26.92% (21/78) for 2 months to 6 months, 15.38% (12/78) for 7 months to 1 year, and 1.5 years to 3 years 10.26% (8/78), 7.69% (6/78) of patients within 1 week, and 7.69% (6/78) of patients with more than 3 years of disease duration.

### 4.7. Laboratory tests

The examination of laboratory indices was not described in 12 patients, and the analysis of the relevant indices in 83 patients revealed mainly abnormalities in sUA, ESR, CRP, WBC, BUN, and SCr. sUA levels were described in 79 of 83 patients with lumbar gout. Among them, 62 cases (78.48%) had varying degrees of elevated sUA, and 17 cases (21.52%) showed normal. Thirty-eight cases had ESR values recorded, of which 32 cases (84.21%) had increased ESR; 41 cases had CRP data, of which 36 cases (87.8%) had elevated CRP; 33 cases had WBC data, of which 18 cases (54.55%) had increased WBC count increased; 14 cases had BUN data, of which 8 cases (57.14%) had elevated BUN; 26 cases had SCr data, of which 17 cases (65.38%) had increased SCr levels to varying degrees. In addition, only 2 patients (2.41%) recorded changes in alkaline phosphatase values there, of which 1 was normal and 1 was elevated.

### 4.8. Site of pathogenesis

**Figure 11.**
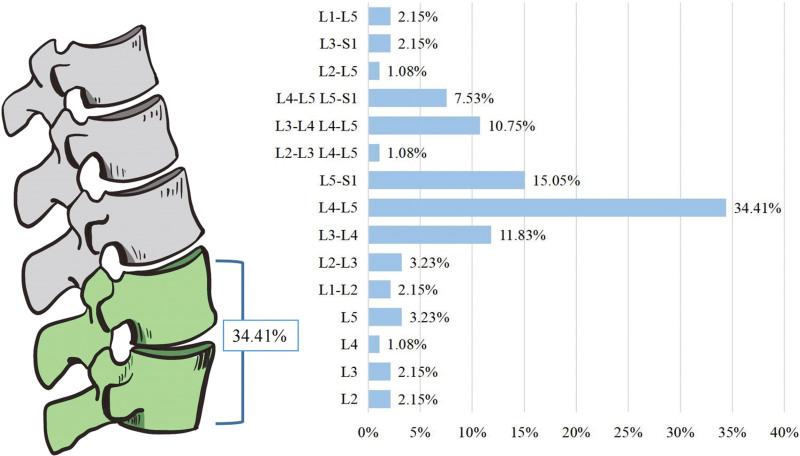
Diagram of incidence site of lumbar gout.

Except for 2 patients in whom the abnormal lumbar spine site was not clearly described, the remaining 93 patients were found to have lesions involving a single vertebral body in 8 cases (8.6%), including 2 cases each of L2 and L3 vertebrae, 1 case of L4 vertebrae, and 3 cases of L5 vertebrae, based on the results of imaging, surgical treatment, and puncture biopsy. Gout involved a single segment in 62 cases (66.67%), L1-L2 in 2 cases, L2-L3 in 3 cases, L3-L4 in 11 cases, L4-L5 in 32 cases, and L5-S1 in 14 cases. The lesions involved 2 segments in 18 cases (19.35%), L2-L3 and L4-L5 in 1 case, L3-L4 and L4-L5 in 10 cases, and L4-L5 and L5-S1 in 7 cases. Three cases (3.22%) involved 3 lumbar segments, including 1 case in L2-L3, L3-L4, and L4-L5 and 2 cases in L3-L4, L4-L5, and L5-S1 (Fig. 11). The high incidence of lumbar gout was predominantly single-segment, with the most common onset at the L4-L5 level, while the lesion site involved the entire lumbar spine in only 2 patients (2.15%).

### 4.9. Treatment

One patient died of acute pulmonary edema after autopsy findings of gout involving the L2 vertebral body, no specific treatment was described in 5 cases, and 89 cases received appropriate treatment. 75.28% (67/89) received surgical treatment such as laminectomy and decompression, mass removal, nerve root release, an intervertebral fusion, and 3 cases (4.48%) were treated by minimally invasive decompression, 1 case (1.49%) was treated via an anterior L5/S1 vertebral body was partially resected, and titanium mesh was placed, and lumbar interbody fusion was performed in 13 patients (19.40%), aged 26 to 71 years, with a mean age of (52.92 ± 13.80) years.

In addition, 35 cases (52.24%) used postoperative drugs in conjunction with anti-inflammatory, analgesic, and uric acid control; 65.71% (23/35) used allopurinol, 54.23% (18/35) used colchicine, 20% (7/35) used non-steroidal anti-inflammatory drugs (NSAIDs), 17.14% (6/35) used glucocorticoids, and 11.43% (4/35) used febuxostat, 2.86% (1/35) used benzbromarone, 2.86% (1/35) used sodium bicarbonate tablets, 2.86% (1/35) used morphine sulfate, 2.86% (1/35) used mannitol, and 2.86% (1/35) used dapsigargin. Twenty-two patients (24.72%) received only non-surgical treatment. Two patients (9.1%) opted for local puncture and drug injection, 1 local injection of prednisolone with the IL-1 receptor antagonist Anakinra, and 1 underwent epidural puncture and glucocorticoid injection with allopurinol; 60% (12/20) used colchicine, 45% (9/20) used glucocorticoids, 45% (9/20) used allopurinol, 30% (6/20) used NSAIDs, 5% (1/20) used febuxostat, 5% (1/20) used Anakinra, 5% (1/20) used sodium bicarbonate tablets, 5% (1/20) used vancomycin, and 5% (1/20) used cefepime.

### 4.10. Prognosis and follow-up

Of the 67 patients treated surgically, 9 (13.43%) patients did not describe specific prognosis and regression. Unfortunately, 2 (2.99%) patients died due to postoperative infection, 56 (83.58%) patients had significant relief of clinical discomfort and different degrees of improvement in neurological dysfunction after surgical treatment, and 32.14% (18/56) were followed up after discharge, ranging from 6 weeks to 2 years, obtaining the ideal therapeutic effect. 22 conservatively treated patients, 17 (77.27%) had significant symptom relief, 1 patient (4.55%) had poor compliance and was unfortunately lost to follow-up, 1 case (4.55%) had no clearly described prognosis, and 3 patients (13.64%) had poor treatment outcomes. In particular, a 25-year-old male developed hypercreatinemia due to recurrent gout attacks and poor renal function, requiring dialysis treatment.

## 5. Discussion

In this case, the patient had obvious symptoms of lumbar pain with numbness in the left lower limb, which is a typical clinical manifestation of nerve root compression, and a preliminary diagnosis of lumbar disc herniation was made. MRI of the lumbar spine suggested an occupying lesion at the L4-L5 level, and the imaging department suggested the possibility of neurogenic tumors and arachnoid cysts, suggesting enhanced examination, but combined with the patient’s 2-year history of gouty arthritis and significantly increased blood uric acid level, lumbar gout was considered, and surgery and pathology confirmed the preoperative diagnosis. Based on this, we also searched the database for literature on gout affecting the lumbar spine and found that the incidence of lumbar gout was indeed relatively low, with only 95 patients reported in the past, including 71 males and 24 females, with a male to female ratio of 2.96:1, and obesity, metabolic diseases, and unhealthy dietary habits may induce risk factors for this disease. The highest age of the patients was 87 years old, and the youngest age was 16 years old, with a large age span of incidence, while the peak incidence was between 56 and 65 years old, which accounted for 27.73% of all cases. Analysis of the patients’ clinical symptoms revealed a predominance of lumbago, backache, fever, and varying degrees of neurological dysfunction, while lumbago was the primary cause of concern and prompted the patients to seek medical attention, and 20% of patients presented with varying degrees of fever, which may be related to a local immune inflammatory response,^[5]^ and should be distinguished from brucellosis spondylitis, lumbar spinal tuberculosis, intervertebral space infection, and osteomyelitis. A history of gout attacks or hyperuricemia in 61.05% of patients, obesity, metabolic diseases, and unhealthy dietary habits may trigger risk factors for gout, while 28.42% of patients had hypertension in combination, indicating a close association with the development of cardiovascular disease.^[6]^ Involvement of other sites was also observed in some patients, and interestingly, the most frequently involved site was the ankle, followed by the wrist, rather than the first metatarsophalangeal joint as we traditionally perceive it.^[7]^

The diagnosis of lumbar gout is difficult due to the deep location of the lesion and the absence of gout nodules on the body surface, which usually do not have the typical clinical manifestations of peripheral gouty arthritis and need to be differentiated from a variety of diseases.^[8]^ X-ray is not specific for the diagnosis of spinal gout, and some patients may present with positive signs at a later stage, but it is generally later than the clinical presentation and is of no significant value for clinical diagnosis. CT can show the destruction of bone and contribute to the identification of the lesion site, but it is of little significance for the differentiation of calcified gout nodulesp.^[9]^ MRI is a standard clinical test, but it is difficult to differentiate from spinal tuberculosis, infection and tumors because of its poor specificity.^[10]^Dual-energy computed tomography (DECT) imaging technique can clearly show urate crystalline deposits and has a high detection rate in patients with arthralgia and suspected gout, especially in the early stages of gout,^[11]^ with a better overall diagnostic performance. However, its diagnostic value still needs to be further verified because it is not yet widespread and is less used in the diagnosis of spinal gout, especially in the differentiation from calcium pyrophosphate deposition disease, and pathological biopsy should still be the gold standard at present. While the high incidence of the disease is mainly in single segment, the onset of the disease is most common at the L4-L5 level, accounting for 34.41% of all patients. In addition, abnormalities in laboratory indicators such as sUA, ESR, CRP, and WBC are of value in the diagnosis of lumbar gout, and changes in BUN and SCr levels may occur with late involvement of the kidneys. We analyzed the laboratory data of 95 patients and found that 78.48% of patients had varying degrees of elevated sUA, 84.21% had increased ESR, 87.8% had elevated CRP, and 54.55% had increased WBC counts. However, ESR, CRP, and WBC are inflammatory indicators which are not very specific in the diagnosis of gout and can only be used as a reference.^[12]^

Of the 89 patients who received the corresponding treatment, 67 patients underwent surgical treatment, including laminectomy decompression, mass removal, screw fixation, intervertebral fusion, etc. There are various clinical options available, but the respective indications must be strictly adhered to, and the best individualized treatment plan must be chosen according to the degree of the patient’s condition and overall situation. Among patients who underwent laminectomy decompression, 19.40% underwent lumbar fusion, and considering that general decompression surgery is not necessary for fusion,^[13]^ it is worth pondering whether patients with lumbar gout need lumbar fusion. It should be objectively prudent to consider the destruction of vertebral bone by gout and the scope of surgical resection reasonably selected to maintain the stability of the lumbar spine and provide for the recovery of spinal cord and nerve root function. For lumbar gout, whether surgery or conservative therapy is chosen, control of uric acid levels should be used throughout the treatment of the disease. There is nothing special in the pharmacological treatment of lumbar gout; in the acute phase of the attack, anti-inflammatory analgesia is its main treatment principle, and glucocorticoids or NSAIDs can be chosen for treatment, together with colchicine, and local injection of glucocorticoids can also be considered to relieve clinical symptoms.^[14,15]^ For postoperative or intermittent, or chronic patients, allopurinol, febuxostat, and benzbromarone can be used routinely to reduce uric acid levels, especially allopurinol is the first-line clinical drug of choice,^[16]^ and it is worth noting that caution should be exercised in the use of such drugs if renal insufficiency is present.

## 6. Conclusion

With this study we can find that gout affecting the Axial skeleton is rare, and a unified and standardized diagnosis and treatment system has not yet been formed, which is why this disease is easily misdiagnosed in clinical practice. Therefore, for patients with lumbar pain, significant neurological dysfunction, or occupying lesions in the vertebral canal, clinicians should take a careful history and systematically and comprehensively analyze and judge the condition by combining symptoms, signs, and ancillary examinations avoid missed diagnosis and misdiagnosis. If a patient has a high uric acid level or a history of gout attacks, the possibility of lumbar gout should be considered. To clarify, the patient should be differentiated from degenerative diseases of the lumbar spine, tuberculosis, tumor, infection, brucellosis, psoriatic spondylolisthesis, psoriatic spondyloarthropathy the diagnosis and make an early intervention.

## Author contributions

Kai Wang: mainly manuscript writer. Quan-Zeng Yang: mainly manuscript writer. Hao-Nan Wen: Text processing and modification. Yun-Xiang Hai: Text processing and modification. Min Song: Subject Guidance and Overall Design. Guo-Dong Gao: Case Operator

**Data curation:** Kai Wang.

**Methodology:** Guo-Dong Gao, Min Song.

**Supervision:** Min Song.

**Writing** – original draft: Kai Wang.

**Writing – review & editing:** Kai Wang, Quan-Zeng Yang, Hao-Nan Wen, Yun-Xaing Hai, Min Song.

## References

[1] Narang RK Topless R Cadzow M. Interactions between serum urate-associated genetic variants and sex on gout risk: analysis of the UK biobank. Arthritis Res Ther. 2019;21:2–9. 30626429 10.1186/s13075-018-1787-5 PMC6327586

[2] Luo H Fang W Zuo X. Analysis of clinical characteristics, diagnosis and treatment of gout patients in China. Chin J Intern Med. 2018;57:27–31. 10.3760/cma.j.issn.0578-1426.2018.01.005 29325307

[3] Hou LC Hsu AR Veeravagu A. Spinal gout in a renal transplant patient: a case report and literature review. Surg Neurol. 2007;67:65–73; discussion 73. 17210304 10.1016/j.surneu.2006.03.038

[4] Kersley GD Mandel L Jeffrey MR. Gout an unusual case with softening and subluxation of the first cervical vertebra and splenomegaly result of acth administration and eventual post-mortem findings. Ann Rheum Dis. 1950;9:282–304. 14800242 10.1136/ard.9.4.282 PMC1011667

[5] Qin DA Song JF Li X-F. Tophaceous gout of lumbar spine with fever mimicking infection. Am J Med. 2018;131:e353–6. 29730358 10.1016/j.amjmed.2018.04.021

[6] Singh JA Gaffo A. Gout epidemiology and comorbidities. Semin Arthritis Rheum. 2020;50:S11–6. 32620196 10.1016/j.semarthrit.2020.04.008

[7] Scuiller A Pascart T Bernard A. La maladie goutteuse [Gout]. Rev Med Interne. 2020;41:396–403. 32201015 10.1016/j.revmed.2020.02.014

[8] Ng W Sin CH Wong CH. Unusual presentation of spinal gout: 2 cases report and literature review. J Orthop Case Rep. 2017;7:50–4. 10.13107/jocr.2250-0685.946 PMC5868884 29600211

[9] Jin HJ Son ES Kim DH. The frequency of axial deposition in Korean patients with gout at a tertiary spine center. Front Med (Lausanne). 2020;7:339. 32850877 10.3389/fmed.2020.00339 PMC7419467

[10] Mogensen MA DeConde RP Sarikaya B. Spinal gout: imaging and clinical features. PM R. 2021;13:1304–6. 34435750 10.1002/pmrj.12699

[11] Singh JA Budzik JF Becce F. Dual-energy computed tomography vs ultrasound, alone or combined, for the diagnosis of gout: a prospective study of accuracy. Rheumatology (Oxford). 2021;60:4861–7. 33410491 10.1093/rheumatology/keaa923

[12] Chowalloor P Raymond WD Cheah P. The burden of subclinical intra-articular inflammation in gout. Int J Rheum Dis. 2020;23:661–8. 32107861 10.1111/1756-185X.13811

[13] Reid PC Morr S Kaiser MG. State of the union: a review of lumbar fusion indications and techniques for degenerative spine disease. J Neurosurg Spine. 2019;31:1–14. 31261133 10.3171/2019.4.SPINE18915

[14] Ko KH Huang GS Chang WC. Clinical images: lumbar spondylolisthesis caused by tophaceous gout. Arthritis Rheum. 2009;60:198. 19116898 10.1002/art.24144

[15] Samuels J Keenan RT Yu R. Erosive spinal tophus in a patient with gout and backache. Bull NYU Hosp Jt Dis. 2010;68:147–8. 20632992

[16] FitzGerald JD Dalbeth N Mikuls T. 2020 American college of rheumatology guideline for the management of gout. Arthritis Care Res (Hoboken). 2020;72:744–60. 32391934 10.1002/acr.24180 PMC10563586

